# An mHealth App for Fibromyalgia-like Post–COVID-19 Syndrome: Protocol for the Analysis of User Experience and Clinical Data

**DOI:** 10.2196/32193

**Published:** 2022-02-04

**Authors:** Marc Blanchard, Lars Backhaus, Pedro Ming Azevedo, Thomas Hügle

**Affiliations:** 1 Department of Rheumatology University Hospital of Lausanne University of Lausanne Vaud Switzerland

**Keywords:** post–COVID-19 syndrome, COVID-19, SARS-CoV-2, mobile health, application, user experience, testing, user interface, long-covid syndrome, mHealth, app, user interface, protocol, reinforcement, learning, strategy, symptom, outcome, patient-reported outcome, therapy, rehabilitation, monitoring

## Abstract

**Background:**

Post–COVID-19 syndrome, also referred as “long covid,” describes persisting symptoms after SARS-CoV-2 infection, including myalgia, fatigue, respiratory, or neurological symptoms. Objective symptoms are often lacking, thus resembling a fibromyalgia-like syndrome. Digital therapeutics have shown efficiency in similar chronic disorders such as fibromyalgia, offering specific disease monitoring and interventions such as cognitive behavioral therapy or physical and respiratory exercise guidance.

**Objective:**

This protocol aims to study the requirements and features of a new mobile health (mHealth) app among patients with fibromyalgia-like post–COVID-19 syndrome in a clinical trial.

**Methods:**

We created a web application prototype for the post–COVID-19 syndrome called “POCOS,” as a web-based rehabilitation tool aiming to improve clinical outcomes. Patients without organ damage or ongoing inflammation will be included in the study. App use will be assessed through user experience questionnaires, focus groups, and clinical data analysis. Subsequently, we will analyze cross-sectional and longitudinal clinical data.

**Results:**

The developed mHealth app consists of a clinically adapted app interface with a simplified patient-reported outcome assessment, monitoring of medical interventions, and disease activity as well as web-based instructions for specific physical and respiratory exercises, stress reduction, and lifestyle instructions. The enrollment of participants is expected to be carried out in November 2021.

**Conclusions:**

User experience plays an important role in digital therapeutics and needs to be clinically tested to allow further improvement. We here describe this process for a new app for the treatment of the fibromyalgia-like post–COVID-19 syndrome and discuss the relevance of the potential outcomes such as natural disease course and disease phenotypes.

**International Registered Report Identifier (IRRID):**

PRR1-10.2196/32193

## Introduction

### Post–COVID-19 Syndrome

Post–COVID–19 syndrome is an increasingly recognized symptom complex occurring after SARS-CoV-2 infection, which has also been called “long COVID-19” [1]. It can be described as persisting organ damage; for example, after intensive care treatment for respiratory failure or more frequently by persisting general unspecific symptoms such as fatigue, myalgia, concentration, and sleep disturbance [2]. Owing to its neurotropism, SARS-CoV-2 can cause neural damage and persisting neurologic symptoms including olfactory dysfunction, neuropathic pain, and transient memory loss [3-5]. Objective findings in imaging or functional testing are classically missing. As an example, there is no typical neurological manifestation induced by COVID-19 on magnetic resonance imaging, but a wide range of different patterns are observed in patients [6]. Thus, the diagnosis of post–COVID-19 syndrome is clinical and difficult to monitor [7]. Similar postviral features have also been attributed in the past to other infectious outbreaks; for example, Epstein-Barr Virus or Q fever [8,9]. After the severe acute respiratory syndrome outbreak in 2003, approximately one-third of the infected patients developed reduced tolerance to exercise many months later despite having normal lung function [10]. In the past, postviral myalgic encephalomyelitis (ME) has been considered a synonym for chronic fatigue syndrome [11]. Patients with ME have also experienced neurovegetative and cognitive dysfunction, often fulfilling the classification criteria for fibromyalgia [12]. It remains unclear whether this dysfunction is a stress-related response of the host, or if it occurs owing to ongoing viral replication [13].

The prevalence of post–COVID-19 syndrome is unclear, but it has been already discussed in the literature as an upcoming relevant health problem, for which therapeutic solutions and scalable health care models must be developed [14,15]. The incidence of post–COVID-19 syndrome after SARS-CoV-2 infection is estimated to be 10%-35%. This estimation can reach 85% for hospitalized patients [16]. In this study, we focus on fibromyalgia-like post–COVID-19 syndromes, albeit without ongoing inflammatory activity or objective organ damage.

### Digital Therapeutics

The use of medical apps as diagnostic but also therapeutic tools is rapidly increasing, fostered not only by the current pandemic but also by the growing acceptance of mobile health (mHealth) [17-19]. Change in legislation in different countries also permits the prescription of therapeutic medical apps, such as Digital Health Applications (DIGAs) in Germany. DIGAs are digital therapeutic tools that meet high quality standards and proof of clinical benefits. They are officially registered and most of them provide tools for cognitive behavioral therapy, exercise instructions, and lifestyle modifications (habits, nutrition, meditation, etc) [20,21].

The pandemic accelerates the development of digital solutions. The United States and Australia have, for example, established remote care systems for patients with chronic diseases and COVID-19 [22]. Surveys developed in Ontario (Canada) between February and May 2020 showed an increase from approximately 1000 clinical-to-patient video calls per day to 14,000, especially concerning elderly patients [23]. These examples illustrate the progressive implementation of digital therapeutics in the modern society. According to several studies, this new field will become a turning point in global health. Unfortunately, most of these studies also indicate that there is a scarcity of qualitative data in this domain [24-26]. The lack of knowledge in user experience (UX) and other factors such as design or gamification, which could influence consumer engagement, may be considered to facilitate the development of digital health solutions [27]. Notwithstanding the trend toward digital therapeutics, it is evident that more research on designing these apps for optimal usability is required [28].

Many currently existing apps offer the technical requirements but lack protocols for user interface (UI) design [29]. Furthermore, clinical study design including therapeutic apps require specific consideration; for example, where and how therapeutic modules are integrated or how patient-reported outcomes (PROs) can be monitored [30].

### UX

UX experiments are key to improve the ergonomics and usability of an app. They occur during the development of the user interface and aim to optimize its usability. UX experiments also help the developer to assess the user’s expectations. They are vital in the process of building a suitable app [31].

UX research on therapeutic medical apps is a growing field as UX interferes with adherence and potentially also with clinical outcomes. However, there is a lack of qualitative research on health and medical apps in general [29]. So far, a combination of three theory models for assessing UX for patients with chronic conditions has been postulated: the Technology Acceptance Model (TAM), the Health Information TAM (HITAM), and Health Belief Model [32]. The TAM measures how users accept technology. The HITAM adds to the concepts in the TAM by incorporating the Health Belief Model [32].

### Aim and Research Questions

In this study, we aim to understand how patients with fibromyalgia-like post–COVID-19 syndrome might use digital therapeutics at the example of the app “POCOS” and how their experience could be improved in regard to onboarding, data entry, data processing, and illustration as well as therapeutic applications. Furthermore, we aim to translate these findings in the development of an optimized clinically adapted app interface, and we speculate on the potential use of such a device for a better scientific understanding of the post–COVID-19 syndrome. By the term “clinically adapted,” we are referring to a patient-centered app architecture tailored to disease-specific needs but also comprehensible and interoperative for clinicians.

The research questions of this study are as follows: 

#### User Experience

- Which symptom or intervention features are considered most important by patients with post–COVID-19 syndrome?
- What is the best way to illustrate disease activity and symptoms?
- Which general therapeutic (medical and paramedical) interventions are considered most useful by patients?
- How often will they use the app over time? What are the best intervals for assessing PROs?
- How useful are online instructions and educational videos?
- What type of therapeutic modules are considered most useful by the patients?
- How useful is a personalized web-based therapy program based on symptom profile and disease activity?

#### Clinical Outcomes

- What is the natural course of post–COVID-19 syndrome? Can phenotypes be identified?
- What are the risk factors for poor subjective or objective outcomes (doctor’s visits or hospitalization)?
- How useful are validated fibromyalgia activity scores, such as the widespread pain index or symptoms severity score for post–COVID-19 syndrome [33]?

## Methods

### UI Implementation

**Figure 1:**
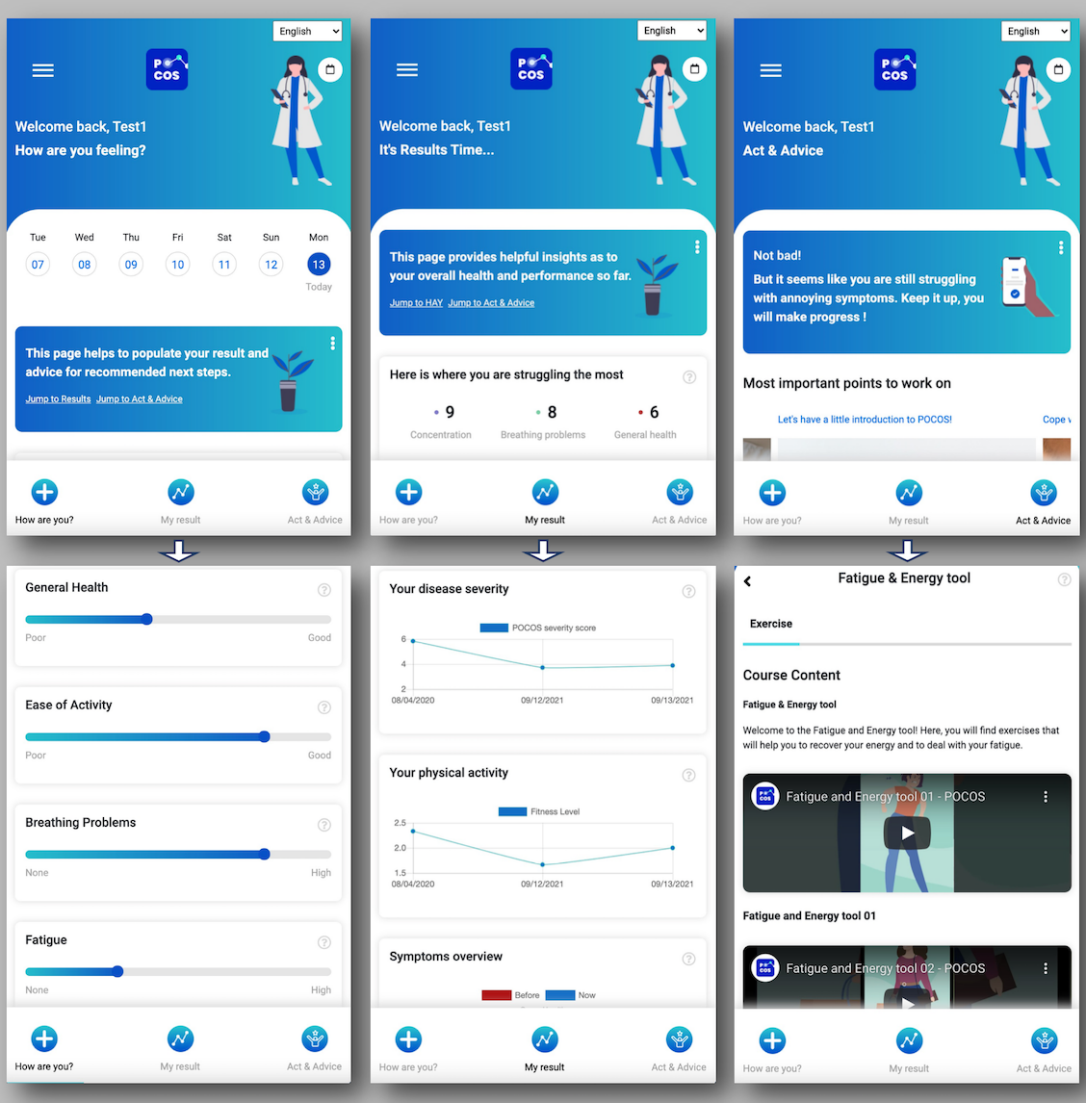
POCOS user interface. Left panel: the “How are you?” screen with electronic patient-reported outcomes and activity. Center panel: the “My result” screen with monitoring of symptom activity and health conditions. Right panel: the “Act & Advice” screen with a personalized training program adapted to the user’s symptoms.

The web application prototype investigated here is called “POCOS,” which stands for post–COVID-19 syndrome. For this purpose, we developed a simplified app interface adapted to assess and treat post–COVID-19 symptoms (Figure 1). The main architecture of this interface is based on different sections resembling a doctor’s visit and disease-specific requirements, respectively:

- Assessment of PROs in accordance with the literature [34].
- Medical and paramedical interventions since last data entry (physiotherapy, laboratory analysis, physical activity, and nutritional aspects).
- Monitoring and visualization of symptom courses and personalized fibromyalgia-like post–COVID-19 disease activity scores.
- Web-based therapy modules with animated videos for information, different exercise instructions (physical, respiratory, smelling, etc), muscle relaxation, stress reduction, cognitive behavioral therapies, mindfulness, as well as exchange with peers and health care professionals. The web-based treatment program will be composed in accordance with the leading disease features: fatigue, stress, pain, sleep problems, respiratory problems, neurologic symptoms, etc. It will also include information about what has helped other patients with similar symptoms recently (“What has helped you the most recently?” output) (Tables 1 and 2).

**Table 1:** Overview of data assessment (onboarding process, patient-reported outcomes, and interventions).

Characteristics		Response type
**Onboarding process (only once after first login)**		
	Age	Birth date input
	Gender	M/F
	Date of suspected or confirmed COVID-19 infection	Date input
	Type of test, if available	ELISA^a^ or direct testing
	Confinement	Yes/No
	(If confinement) Confinement duration	Number of days
	(If confinement) Type of confinement place	Small/big flat, house, house with garden
	Other infected family members or peers	Yes/No
	Initial symptoms	Multiple choice (to be determined)
	Psychosocial factors	Multiple choice (to be determined)
	Pre-existing disorders	Multiple choice (to be determined)
	Smoking	Yes/No
**Patient-reported outcomes (daily or weekly)**		
	General health	Horizontal slider (0-10 scale)
	Breathing problems	Horizontal slider (0-10 scale)
	Overall pain	Horizontal slider (0-10 scale)
	Tender points	Clickable on body diagram (frond and back)
	Memory level	Horizontal slider (0-10 scale)
	Fatigue	Horizontal slider (0-10 scale)
	Concentration	Horizontal slider (0-10 scale)
	Ease of activity	Horizontal slider (0-10 scale)
	Smelling and taste problems	Horizontal slider (0-10 scale)
	Mood	Clickable smileys (1-5 scale)
**Interventions (daily or weekly)**		
	Physical activity (walk, sport, and exercises)	Frequency (once, 2-3 times a week, everyday)
	Weight	Numeric
	Number of physician visits since the last input	Never, 1-3, >3
	Have you been in hospital since last input?	Yes/No
	Did you have therapeutic sessions since last input?	Selection (therapy type list, to be determined), frequency
	Pain killers use	Frequency (per week)
	What has helped you the most recently?	Free text entry
	Laboratory values from blood test (if available)	Type of value, value (concentration)
	Current medication	Selection (list of drugs available on the market) and frequency taken

^a^ELISA: enzyme-linked immunosorbent assay.

**Table 2:** Therapy modules, type of content proposed to the patient in each therapy module and corresponding patient-reported outcomes (related patient-related outcomes).

Therapy module	Content types (video/tutorials, images, and text)	Related patient-related outcomes
Respiratory	Respiratory exercises and yoga	Breathing problems, fatigue, ease of activity, and concentration
Relaxation	Yoga, meditation, and physical and relaxation exercises	Breathing problems, fatigue, overall pain, concentration, ease of activity, and mood
Energy	Relaxation exercises, yoga, advice to save energy, and meditation	Fatigue, ease of activity, mood, and overall pain
Pain management	Therapeutic stories to understand pain, yoga, meditation, and relaxation	Overall pain
Memory	Memory exercises, therapeutic stories, and advice to improve memory	Memory level and concentration
Physio	Physical exercises and yoga	Ease of activity, breathing problems, overall pain, and fatigue
Smelling and taste	Smelling and taste exercises, instructions, and self-assessment	Smelling and taste
Mental enhancement	Motivation exercises and positive thinking advice	Mood, concentration, ease of activity, and fatigue

### Inclusion and Exclusion Criteria

As no official definition for the post–COVID-19 syndrome exists, we decided to include the following patients: (1) those with a proven SARS-CoV-2 infection (direct test or enzyme-linked immunosorbent assay) and (2) those with persisting symptoms such as pain, fatigue, sleep disturbance, respiratory symptoms, or concentration problems over 3 months [35].

Currently hospitalized patients and those with objective signs of active infection such as fever, increased C-reactive protein, active other viral or bacterial infection, and active immune-mediated, oncologic, or psychiatric disease are not the focus of this study and will be excluded. We will also exclude patients who are hospitalized in intensive care units or those with past or persisting organ dysfunction.

### Clinical Data Assessment

Age, gender, family situation, education, and information on the type and duration of confinement during the pandemic will be assessed during the onboarding process. We will record initial COVID-19 symptoms as well as pre-existing disorders including depression, nutritional factors, and smoking (Table 1).

PROs will be assessed as listed in Table 1 and have been selected in accordance with the main symptoms of post–COVID-19 syndrome reported in the literature [2]. They are collected on a daily or weekly basis.

On a daily and weekly basis, we will collect information on work activity and sick leave, doctor visits, medication, and hospitalizations. As a further feature, patients can type in what helped them most in the last week to monitor and share with patients with similar profiles (Table 1). The data will be analyzed cross-sectionally and longitudinally to identify phenotypes and to calculate risk models.

Widespread pain index and the symptom severity score will be assessed on the basis of PROs and clickable body diagrams (Table 1) data [33].

### UI/UX Experiments

**Figure 2:**
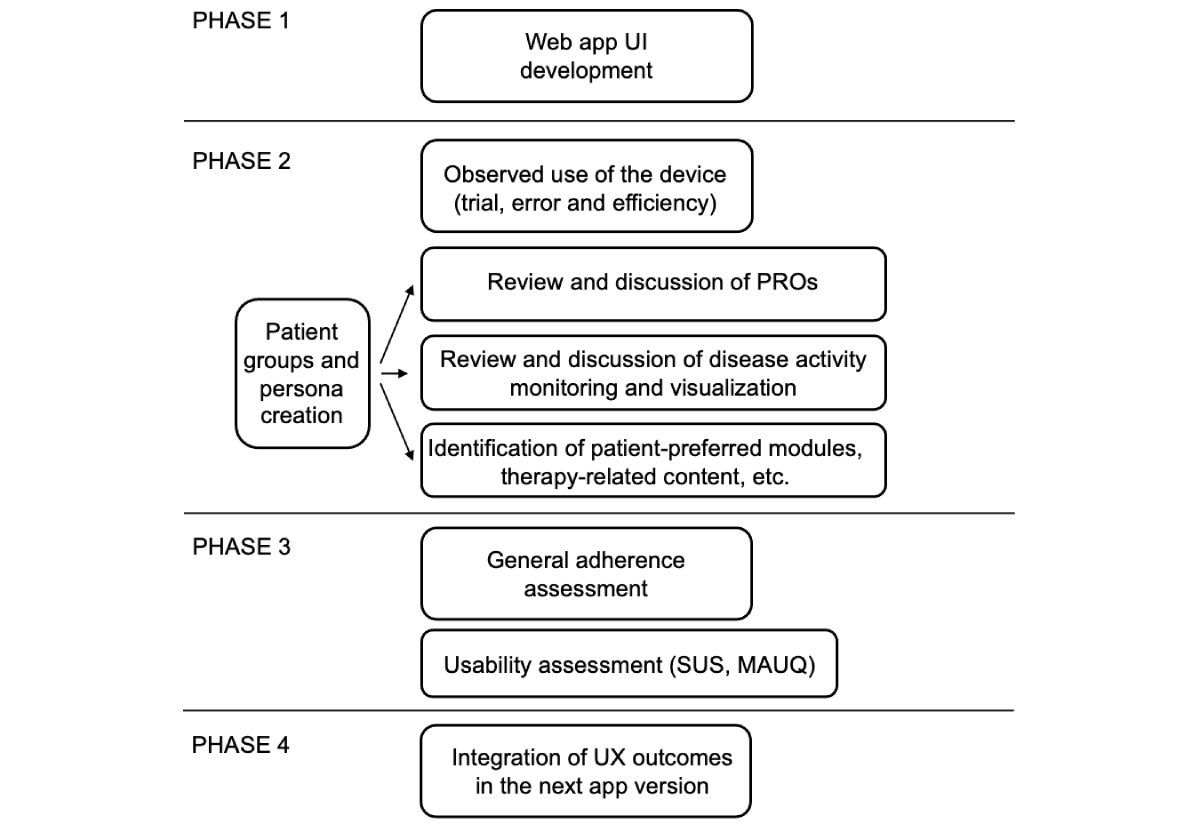
User experience research and the experimental design. MAUQ: mHealth App Usability Questionnaire, PRO: patient-reported outcome, SUS: System Usability Scale, UI: user interface, UX: user experience.

For UI/UX assessment, we aim to examine the interactions between patients with post–COVID-19 syndrome and the “POCOS” app in terms of quality of experience. UX research will be performed by providing POCOS as an app prototype. The following analyses will be performed (Figure 2): (1) interviews with patients with post–COVID-19 syndrome groups and creation of personas (clustering patient groups by similarities); (2) System Usability Scale (SUS) [36], mHealth App Usability Questionnaire (MAUQ) [37], and the Brief Medication Questionnaire; and (3) observed use of device (task success rate, errors, efficiency, and time spent).

### Patient and User Groups

The patients will be selected after obtaining ethical approval. Owing to the changing and diverse nature of diseases, we will further have user personas for each group to test our findings and possible hypothesis. Personas have proven to be a crucial factor when designing user tests through several research. In their research for user preferences and persona design in cardiovascular disease, Haldane et al [38] reported that personas are a key feature of user-centered design and are used in many fields from design to marketing and product, and it aims at understanding large and diverse target audiences. Therefore, our study will also focus on studying personas to identify diverse patient groups.

Personas will be created by patients with post–COVID-19 syndrome in accordance with their needs, their habits, and their challenges while using the app. The personas will be developed on the basis of the most common characteristic of the patient groups. The type of persona is also influenced by Haldane et al [38] as they outline a general approach to mHealth user types.

### User Interface Testing

Testing of nonfunctional properties of the app UI, which will lead to nonfunctional app requirements, will be performed. These properties (especially design or esthetics) are likely to affect the users’ experiences and are therefore crucial to investigate.

To decide on the color scheme, font, spacing, alignment, images, videos, and sound, we will perform A/B testing with different patient groups. While group A will use the app in blue, group B will use the app in the green. According to previous analyses, the blue color is preferred for promoting the identity of clinics and hospitals, while the green color represents nature and health [39]. After their use of the app, patients will be asked about their preferences on the visual attributes with a questionnaire on design principles and choices, derived from the qualitative research on color theory. The data gathered will help us identify the color and design choice of different age groups and genders.

Along with the rating screen, charts are used for users to reach and monitor their progress. To provide specific patient groups the opportunity to decide on what they see on their screen, we will be using the Card Sorting Method. Cart Sorting is a method that involves screenshots of each app to be printed on cards and presented to the users asking them to place the cards in the order that they thought most appropriate [40].

### Interaction and Usability

Interaction and usability investigations will result in the functional requirements of the app. These experiments will lead to the eventual modification, suppression, or validation of the app features. These UX outcomes will then be integrated in the subsequent app version.

Patients will be assigned tasks to complete with the app in a given timeframe. These tasks will include but not be limited to *enter today’s mood*, *view chart of the pain*, *go to advices page*, *adjust yesterday’s sleep quality*, *check last week data*, etc. The tasks will be modified in accordance with the prototype design and ready-to-use features. We will also record the patients’ task time to find the optimal speed and which functions seem to take longer for the patients. As they would while using the app on their own, they will be asked to input their symptoms and report on their pain. To complete the task, the user’s choice of interaction will be recorded by logging their errors such as tapping instead of double-tapping while inputting text.

Along with the interaction and usability observation, upon completion of each task, users will be administered the SUS [41]. Originally developed by John Brooke in 1986, the SUS facilitates the evaluation of a wide variety of products and features including hardware, software, mobile devices, and apps. It is a 10-item questionnaire that allows us to understand the efficiency of the system and how easily it can be used by the user. After the data are collected, we will be able to score the system and readdress issues with usability if necessary. After each experiment, we will also ask our patients to provide their opinion on the system along with what they liked and what they thought could be improved.

In addition to the SUS, users will also be administered the MAUQ to fill. This 10-15–item questionnaire is specific for mHealth apps and is commonly used and adapted to measure usability [37].

### Medical Adherence

The importance of user experience in relation to medical adherence is well-documented within the discipline of health psychology. According to Dayer et al [42], low adherence causes approximately 33%-69% of medication-related hospitalizations and US $100 billion in annual health care costs. Currently, there are no tests to analyze app adherence. Currently available mHealth apps including Med Agenda, Dosecast, and MedSimple use push notifications such as “time to take your medication” for adherence. Furthermore, apps such as Med Agenda, RxmindMe, Dosecast, MedsIQ, PillManager, and MediMemory require a box to be ticked when the patients take their medication. All of these apps have very low ratings on both Apple App Store and Google Play as they do not possess the features they claim to have.

To create patient-based adherence, we will create a survey to ask the user how often they would like to be reminded to use the app (and to provide the PROs). The Brief Medication Questionnaire [43] will be used to explore both patients’ medication-taking behavior and barriers to adherence. It has a 5-item “Regime” screen, a 2-item “Belief” screen, and a 2-item “Recall” screen. These screens assess how patients took their medications in the past week, the effectiveness of the drug, and concerning features. The test will help us generate our personas by identifying patients who need assistance with their medications, highlight their concerns, and provide novel insights for improvement and development suggestions.

### Clinical Data Analysis

Cross-sectional and prospective characterization of the post–COVID-19 syndrome is key for its understanding and treatment. The POCOS app will collect information on initial COVID-19 infection and subsequent symptoms over time. PROs and medical and paramedical interventions including doctor’s visits and hospitalization will be assessed on at least a weekly basis.

Power calculation: at the beginning of 2021, a total of 33,000 individuals survived a SARS-CoV-2 infection in Switzerland. We estimate that 20%-30% of the patients after a SARS-CoV-2 infection experience marked fatigue and reduced quality of life.

Sample size consideration: data will not be analyzed in order to prove certain hypothesis (exploratory data analysis). The minimal required sample size (n) is determined as follows:

*n ≥ N + (N − 1) × є 2 z 2 × p × q*
**(1)**

where *N* is the number of patients who survived a SARS-CoV-2 infection in Switzerland, 𝜀 is the error accuracy (tolerated error), *z* is the quantile of the standard normal distribution, *p* is the relative frequency of people with post–COVID-19 syndrome (fatigue of at least >3 months), *q*= 1 – *p*. In this case, N=33,000, є=5%, *z*=1.96, *p*=0.15, and *q*=0.85; thus, a minimal sample size of 195 individuals is required. A high dropout rate of 25% is assumed, so that a total of 250 patients who survived a SARS-CoV-2 infection will be initially included in the study. Explorative analysis will be performed with cross-tabs and correlations to identify associations. Repeated measurements analyses will be performed to explore multivariate associations between baseline status (adjusting for age, sex, and disease duration) and disease status after 3 or 6 months. Furthermore, logistic regression analysis will be used to analyze multivariate associations among binary endpoints (eg, achievement of remission, myalgia [yes/no], and work incapacity [yes/no]) at 6 and 12 months. We will perform logistic regression analyses adjusting for age, sex, and BMI to identify symptoms at acute infection (anosmia, fever, persistent cough, fatigue, shortness of breath, diarrhea, chest pain, and hoarse voice), which might be associated with the development of post–COVID-19 syndrome. The aim is to set up a model to predict poor outcomes. At a later step, with higher numbers of included individuals, supervised machine learning using labeled data will be used to generate prediction models, feature importance, and heat maps based on classification and regression analyses.

### Ethical Considerations

The study proposal has been conditionally accepted by the regional ethical committee. This study is expected to commence in November 2021.

## Results

The provisory frond-end development (UI) of the POCOS app has been completed in the form of a clinically adapted architecture (Figure 1). The back-end development is ongoing, especially at the level of data management. A landing website and an introduction video for the app have been prepared. The therapeutic content (videos, articles, and other type of media) has been uploaded on the app. A fully functional version of the app (including web and mobile versions) is expected for the end of September 2021.

## Discussion

### Expected Findings

We here demonstrate UI development of a new mHealth app for post–COVID-19 syndrome, which focuses on the fibromyalgia-like phenotype. The decision for this phenotype was based on its frequency and difficulty to manage symptoms, taking into account the lack of objective clinical signs and biomarkers. Furthermore, we previously developed and used digital support tools for patients with fibromyalgia and chronic fatigue syndromes at our center. As post–COVID-19 syndrome shares clinical similarities with other common chronic syndromes (fibromyalgia, chronic pain syndrome, chronic fatigue syndrome, and rheumatoid arthritis), this study may also provide insights into interesting elements for digital therapeutics that are applied for other chronic diseases [44-47].

The collection of clinical post–COVID-19 data is crucial for its understanding and symptom management. Potentially, data obtained by this app will facilitate the identification of prognostic markers and markers for disease activity, respectively. A strong point of this protocol is the development of the advanced UI frontend including therapy-related content. Therefore, in an optimized version based on the results obtained from this study, we expect a quick and high user interest.

### Limitations

As a limitation, PROs and disease activity markers used in this app have not been validated for the post–COVID-19 syndrome. We would also like to emphasize that this protocol will not provide information on efficacy or safety of this app. In any case, the therapeutic value of this app will have to be tested in a future validation study.

### Conclusions

In conclusion, this study will provide new and potentially large-scale information on the outcomes of patients with post–COVID-19 syndrome. The focus of this protocol on UI/UX design will potentially improve the knowledge on interactions between patients and an mHealth interface.

## Abbreviations

DIGA: Digital Health Applications
HITAM: Health Information Technology Acceptance Model
MAUQ: mHealth App Usability Questionnaire
ME: myalgic encephalomyelitis
mHealth: mobile health
SUS: System Usability Scale
TAM: Technology Acceptance Model
UI: user interface
UX: user experience
